# Climate‐Resilient Agriculture Practices for Enhancing Resilient Practices and Food Systems in Dry Regions

**DOI:** 10.1002/pei3.70116

**Published:** 2026-01-11

**Authors:** Andrew Tapiwa Kugedera, Bhukya Sri Sai Siddartha Naik

**Affiliations:** ^1^ Department of Agriculture and Research Morgenster Teachers College Masvingo Zimbabwe; ^2^ Department of Agronomy, Agricultural College, Warangal Professor Jayashankar Telangana Agricultural University Warangal Telangana India

**Keywords:** agroforestry, climate change, climate resilient, crop rotation, smallholder farmers

## Abstract

Dry regions are primarily inhabited by smallholder farmers who have limited capacity to enhance agricultural productivity, particularly in crop production. These areas are characterized by low and erratic rainfall that does not support crops to maturity. This underlying study has been compiled by using a systematic review of papers published between 2020 and 2025. A total of 1200 papers were selected and screening was done to remove 790 duplicates, 195 had no proper information about climate‐smart agriculture (CSA), and 139 were published before 2020, leaving a total of 76 papers included in the study. The primary objective of this systematic review was to explore resilient agricultural technologies suitable for dry regions to improve food systems. Resilient agricultural practices suitable for dry regions include soil water conservation, irrigation, crop diversification, and cultivating climate‐resilient crops that include sorghum, cowpeas, and millets to enhance the food system. Growing climate‐resilient crops is regarded as a key option in drought‐prone areas that improves crop yields and food availability. Combining water management, soil conservation, and sorghum in low rainfall areas increased yield from 200 to 1140 kg ha^−1^ in Zimbabwe and from 250 to 5675 kg ha^−1^ in Kenya. An increase in other crops, such as maize, has also been reported with the use of crop rotation, irrigation systems, and agroforestry. Improvements in food systems reduce hunger and poverty, and empower smallholder farmers in dry regions to enhance their livelihoods. Similarly, examples of climate‐resilient agriculture options have also been presented, giving relevant examples. Smallholder farmers are recommended to adopt climate‐resilient agricultural practices to all farmers to mitigate climate change, reduce food insecurity, and improve rural livelihoods.

## 1 Introduction

Food security in many developing countries across the world has been hindered by climate change, which contributed to high temperatures, low and erratic rainfall that is unevenly distributed, and outbreaks of pests and diseases. Low and erratic rainfall has been recently noted by smallholder farmers, and this contributed immensely towards food insecurity (Mutengwa et al. 2023; Paul Jr et al. 2023). Dry regions are the most suffering areas since they have already been characterized by low rainfall (< 450 mm per annum), high temperatures (> 30°C), and short growing seasons (80–95 days). The shortening of the cropping season in semi‐arid areas is associated with low and erratic rainfall that delays the onset of rainfall, causes farmers to grow crops late, and rainfall also ends at the end of April, failing to support crops such as groundnuts that require longer cropping seasons. Short cropping seasons are associated with longer dry spells that usually occur at the end of March when flowering crops are in need of water, hence causing a reduction in crop yields. Smallholder farmers in many countries are shifting from traditional farming methods to new technologies that have the potential to increase food systems. Additionally, new farming systems that farmers can adopt must be climate‐resilient agriculture (CRA) that mitigate and adapt to extreme climate change so that communal farming can be improved (Abou‐Hadid 2025; Adimassu et al. 2025). In addition, extreme temperatures (> 30°C) increase transpiration and lower chances of crop maturity, and cause a decline in crop yields (< 1000 kg ha^−1^), and minimizes farm income by more than 100% as some farmers realize total crop failure. Many farmers in smallholder farming systems have limited technical knowledge about CRA and produce low crop yields, that is, less than 1000 kg ha^−1^ for maize (Chiturike et al. 2024), 300 kg ha^−1^ for sorghum and millets (Alemu Tolossa et al. 2025; Feyanbule et al. 2025), and 100 kg ha^−1^ for groundnuts and cowpeas (Nagaravalli 2022). Soil infertility has been caused by inadequate application of nutrient amendments by smallholder farmers who are resource‐poor, although several organizations are promoting the use of CRA practices to improve food systems in arid and semi‐arid regions. A continuous decline in food systems calls for a systematic review to identify various CRA that smallholder farmers can adopt.

Soil fertility has also been affected due to climate change, because heavy storms received in dry regions due to high temperatures cause severe surface runoff and soil erosion that washes away topsoil and exposes subsoils that are nutrient deficient (Guo et al. 2024; Mesele et al. 2025). There is a need to adopt CRA technologies that induce resilience in agriculture and promote crop productivity as well as food security, especially in dry regions that receive low and erratic rainfall (Azadi et al. 2021; Victory et al. 2022; Bo et al. 2023; Zhao et al. 2023; Hussain et al. 2025). Furthermore, smallholder farmers were failing to apply adequate mineral fertilizer of 300–400 kg ha^−1^ of both NPK and Nitrogen fertilizers (Kugedera, Nyamadzawo, and Mandumbu 2022), and less than 20–40 t ha^−1^ organic manure such as cattle manure (Kubiku et al. 2022).

CSA is a technology that farmers can adopt to mitigate and adapt to changing climate, enhance plant productivity, increase food security and resilience in low rainfall areas, and farm profitability (Adimassu et al. 2025). The adoption of CSA practices such as biochar improves soil condition by reducing bulk density in sandy soils from 1.1 to 0.99 g cm^−3^ (Mwadalu 2023). Additionally, use of organic amendments and alley cropping increases water and nutrient retention, reducing surface runoff by mulching, increasing infiltration of rainwater, and enhancing climate‐resilience in crops (Scholz et al. 2014; Bo et al. 2023). In addition, biochar can also reduce the use of synthetic fertilizers and enhance carbon sequestration, minimize the concentration of nitrous oxide (N_2_O), and regulate soil pH because biochar from woody feedstock is alkaline (9.5–12.5) and neutralizes acidity in sandy soils to a pH range of 5.5 to 7.5 (Abhishek et al. 2022; Kugedera et al. 2025). This allows farmers to create a conducive environment for crop productivity. In addition, biochar can be combined with other organic amendments (cattle manure, compost, and vermicompost) to improve soil fertility since it is another major parameter that reduces crop yields and food systems in dry regions. Integration of biochar with vermicompost increased water retention, soil porosity (from 20% to 60%), lower bulk density (from 1.3 to 1.1 g cm^−3^), and enhanced plant growth and yield (Gao et al. 2025). These organic nutrient sources and soil conditioners are important in improving nutrient availability, rehabilitating soil quality, and inducing stress resistance in crops so that they thrive well under a changing climate (Chi et al. 2024; Gao et al. 2025). Other CSA practices that enhance resilience in agriculture include rainwater harvesting (RWH) techniques, agroforestry, conservation agriculture (CA), and growing drought‐tolerant (sorghum, millets, cowpeas, and rye) crops that thrive under harsh conditions (Heinz et al. 2024; Adesogan and Sasanya 2025; Mlambo and Mufandaedza 2025; Swadhi 2025). Moreover, climate‐resilient water management techniques such as tied ridges, drip irrigation, and planting pits can also be used to induce resilience in agriculture (Naqvi et al. 2025).

CSA strategies can be integrated to conserve soil and water, improve crop growth and yield. The integrated approach can foster and create a conducive environment that minimizes soil, water, and nutrient loss, facilitated by the use of water harvesting techniques and organic manure that increase water retention (Kaushal et al. 2021; Didenko 2025; Kubiku et al. 2025). This integration can be a cheaper option to enhance resilience and improve food systems in dry regions. However, the use of CSA is affected by inadequate technical knowledge and poor dissemination of information from researchers. Smallholder farmers have not been well educated about CSA techniques to the extent that they regard them as government initiatives that make them suffer. There is a need to come up with education programs and training systems that equip smallholder farmers with knowledge about CSA and its benefits. Therefore, the primary objective of this systematic review was to unpack various resilient agricultural technologies suitable for dry regions to improve food systems.

## 2 Methodology

**FIGURE 1:**
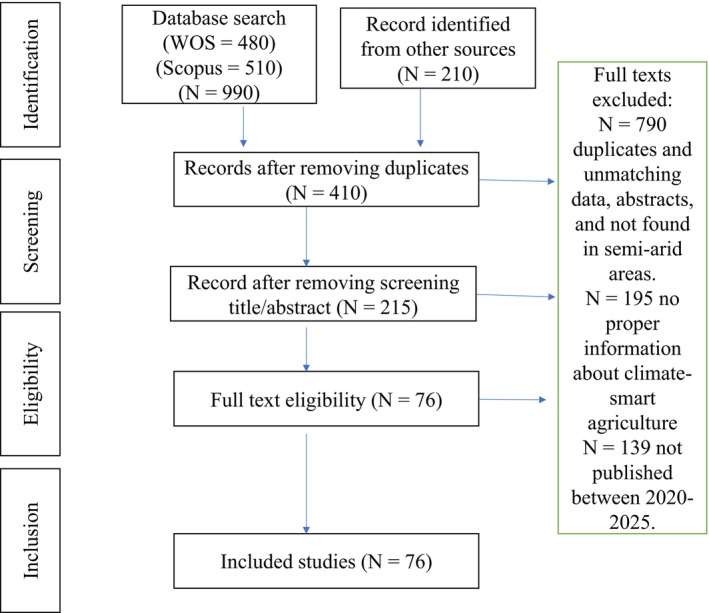
PRISMA flow chart for data identification, screening, eligibility, and inclusion.

The study employed a systematic review, and a literature search conducted across several databases, including Scopus, Elsevier, Google Scholar, ResearchGate, and Web of Science. The search started in April to June 2025. Searching criteria used the following keywords: climate‐smart agriculture, water management, soil resilience, climate resilience, water saving techniques for dry areas, and agroforestry for agriculture resilience, climate‐smart food systems, climate resilience food security, climate‐resilient crops*dry areas, rainwater harvesting* climate‐resilient crops* semi‐arid areas. Titles and abstracts were also used to improve searching criteria. The study adopted the preferred reporting items for systematic reviews and meta‐analysis (PRISMA) where article selection was the first stage. The selection stage produced 1200 articles and screening was done to remove abstracts, duplicates, articles published in predatory journals, and articles not from dry or semi‐arid areas. Screening leaves a total of 130 articles. Further screening was done to remove articles that were published before 2020; only those that carried important information not found in articles published in the 5 years from 2020 to 2025 were left, but they were very few. The study found 76 articles to be eligible and were included as shown in Figure 1. References were generated using Zotero.

## 3 Results and Discussion

### 3.1 Influence of Climate Change on Agriculture

Human activities have increased the concentration of nitrous oxide and carbon dioxide in the atmosphere with an average of 30%–75% in semi‐arid areas, causing a reduction in rainfall chances by 40%–60%, minimizing favorable weather patterns, and increasing temperatures from 25°C to 34°C (Adamides et al. 2020). Rainfall reliability decreases by 20%, distribution patterns, and total amounts received decline by 40%, and contribute to the shortening of the growing season from 120 days to 80–95 days. A shift in prevailing winds that brings in rainfall also occurs, and this lowers chances of rainfall, leading to dry spells of more than 20 days and drought (Abou‐Hadid 2025; Bogale et al. 2025).

Climate change has been reported to reduce crop growth due to long mid‐season droughts that reduce soil moisture from 15% to 5.88% and nutrient uptake, limiting crop yields and food availability by 60%. Furthermore, changing climate also contributes to the outbreak of new pests and diseases that can wipe out crops and cause total crop failure. New pests and diseases take time for researchers to come up with viable solutions, and this reduces crop growth and yields. High temperatures (> 30°C) may also lead to heatwaves that induce increased evaporation rates, wilting, and premature death of crops (Karimi et al. 2024; Dubey et al. 2025). Furthermore, high evaporation rates may lead to total crop failure, which reduces food security in dry regions, mainly semi‐arid areas. Emission of greenhouse gases (GHGs) from irrigations (nitrous oxides), livestock (methane), and agricultural fields due to the use of nitrogenous fertilizers also contribute to climate change, which reduces crop growth, yields, and food systems in arid and semi‐arid areas where smallholder farmers are mainly found. Climate change can also shorten the cropping season by delaying the onset of rainfall, and this also changes growing patterns for smallholder farmers, reducing chances of crops such as finger millet that take 135–140 days to maturity. This reduces the variety of crops grown and limits food variety and availability. In some instances, climate change favors the hatching and multiplication of dangerous pests and pathogens that initially affect crops and reduce yields.

### 3.2 CSA Technologies That Improve Agriculture Resilience and Food Systems

CSA strategies are beneficial to smallholder farmers as they create a win‐win situation that improves crop productivity, enhances resilience in agriculture, and increases food security. These strategies are crucial to adopt as they play a key role in restoring degraded lands, improving soil fertility, increasing nutrient availability, soil organic matter (SOM), and conserving soil moisture. The adoption of CSA strategies such as mulching, cover cropping, intercropping, agroforestry, application of organic amendments, growing climate‐resilient crops, and RWH techniques has the capacity to enhance resilient agriculture, especially for smallholder farmers (Scholz et al. 2014; Azadi et al. 2021; Zhao et al. 2023; Ayilara et al. 2025; Jayakody et al. 2025; Mlambo and Mufandaedza 2025). RWH techniques such as tied contours were reported to increase soil moisture from 4.5% to 10.9%, and raise sorghum grain yield from 450 to 967 kg ha^−1^ in Zimbabwe (Kugedera, Nyamadzawo, and Mandumbu 2022). Organic amendments such as biochar were also reported to increase grain yield of maize from 4500 to 12,300 kg ha^−1^ (Wang et al. 2023). Resilient agriculture is the key strategy in helping communal farmers to fight the effects of low rainfall and soil infertility due to climate change. So, the ministry of agriculture in developing countries can draft policies that can support smallholder farmers to adopt and implement them to increase food systems. Capacity building programs can be done to equip smallholder farmers with technical skills and knowledge about RWH techniques and use of organic amendments.

#### 3.2.1 Drought Tolerant Crops

Drought tolerant crops (sorghum, millets, rye, and cowpeas) perform better under harsh environmental conditions, and they survive climate change vulnerability. These drought tolerant crops have high efficiency in using water and nutrients, and are resistant to pests and disease caused by climate change (Heinz et al. 2024). They thrive under high temperatures (> 30°C), heatwaves, low rainfall (< 450 mm), and nutrient deficiencies in the soil. Dry regions are mainly associated with infertile sandy soils, but climate‐resilient crops that include sorghum and various millets (e.g., proso, barnyard, finger, and pearl) perform better under these conditions (Muruganantham et al. 2025). Finger millet grown in poor soils in India survived well and produced good grain yields (850–1600 kg ha^−1^) that support farmers throughout the year (Nagaraja et al. 2025). In countries like Zimbabwe, finger millet, pearl millet, and sorghum are grown in low rainfall areas (250–400 mm per annum) that are associated with high temperatures, such as Marange, Buhera, Chivi, Gwanda, and Mwenezi (Kugedera, Nyamadzawo, Mandumbu, and Nyamangara 2022). These areas are prone to frequent droughts, but farmers who grow climate‐resilient crops enjoy good harvests and food security. In addition, finger millet grown in Nepal was reported to produce approximately 1650 kg ha^−1^ (Khadka et al. 2018; Gupta et al. 2022), and sorghum grown in Kenya, Tanzania, Uganda, and Zimbabwe produced yields that vary from 450 to 5400 kg ha^−1^ (Desta et al. 2022; Kubiku et al. 2022; Akinseye et al. 2023). Furthermore, climate‐resilient crops are important in inducing food security even during drought periods. Commonly grown climate‐resilient crops in Zimbabwe include sorghum, finger, and pearl millet. Yields of sorghum and millets from semi‐arid regions were in the same range of 450–1900 kg ha^−1^ in Zimbabwe, Kenya, Tanzania, Nepal, Nigeria, Mali, and Uganda (Ebanyat et al. 2021; Kubiku et al. 2022; Kalema et al. 2022; Wainaina 2023; Sharma et al. 2024; Akinseye et al. 2023; Atube et al. 2025; Feyanbule et al. 2025).

#### 3.2.2 Climate‐Smart Fertility Management and Soil Resilience

Soil resilience is the capacity of soil to restore and recover from disturbances and allow it to perform essential functions that support crop productivity. This has the potential of allowing soil restoration after land degradation, returning its fertility by increasing SOM, lowering surface runoff, and increasing water retention (Kaushal et al. 2021). These can be achieved through proper climate‐smart fertility and nutrient management. Climate‐smart nutrient management enhances crop productivity in the wake of an increasing human population under a changing climate (Naskar et al. 2025). Nutrient availability is affected by an ever‐increasing temperature that reduces soil moisture and minimizes nutrient absorption. Climate‐smart nutrient management allows optimization of nutrients in the soil, allowing crops to absorb them and boost crop growth. Nutrient management also helps in reducing emissions of GHGs, and this mitigates climate change as well as enhances climate change adaptation in agriculture (Jatav and Rajput 2025). This enhances resilient agriculture and promotes productivity, especially in areas dominated by sandy soils that are inherently fertile. In addition, farmers can use biochar as a climate‐smart soil fertility management and soil resilience option that conditions the soil, improves availability of nutrients, regulates soil pH, lowers bulk density (from 1.1 to 0.99 g cm^−3^), increases water and nutrient retention due to increased total soil porosity and organic matter that minimize leaching and lower bulk density (Abhishek et al. 2022; Gao et al. 2025; Kugedera et al. 2025; Liang et al. 2025).

Furthermore, biochar has been reported to increase crop productivity in low rainfall areas as a means of improving food systems in semi‐arid areas (Choudhary et al. 2021; Bo et al. 2023; Ahmad et al. 2024). Moreover, smallholder farmers can adopt organic manure to boost crop yields in marginalized areas. Marginalized areas are associated with sandy soils that need to be improved in terms of structure, nutrient retention, SOC, and SOM. So, the use of animal manure, vermicompost, and compost has the potential of increasing soil resilience and WUE from 2.1 to 6.89 kg ha^−1^ mm^−1^ for maize and sorghum, NUE from 2.4 to 13.6 kg kg^−1^, infiltration rates, water retention, and nutrient availability that promote food security through enhanced crop growth and yields (El‐Shazly et al. 2023; Bai et al. 2025; Gao et al. 2025). The use of these organic amendments reduces emission of GHGs, promotes carbon sequestration, and improves crop productivity by 50%–200% especially in sandy soil where farmers grow climate‐resilient crops that include finger millet and sorghum (Mwadalu et al. 2022; Wainaina 2023; Feyanbule et al. 2025). If smallholder farmers promote organic manure, soil health and quality can be improved and reduce the use of mineral fertilizers than contribute to climate change.

#### 3.2.3 Agroforestry Systems

Agricultural productivity has been declining over the years as a result of changing climate, which contributed to reduced soil health, moisture, and nutrient stress. This can be solved with incorporating agroforestry practices that enhance resilience in agriculture and improve productivity. Hedgerow cropping causes foliage to accumulate on the soil surface, acts as a mulch, and conserves soil moisture by preventing direct heating of on soil surfaces (Mantino et al. 2023). In addition, a mulch layer also reduces surface runoff by 60%, soil erosion by 75%, and increases nutrient recycling through decomposition of organic matter. This increases soil fertility, availability of nutrients, and moisture that allow crop growth, and increases yields from 250 sorghum to 1200 kg ha^−1^ (Mantino et al. 2023). This creates a strong resilience of crops to a changing climate, such as high temperatures, since trees in alley cropping provide shade that reduces the effects of heat waves (Ayilara et al. 2025). Furthermore, agroforestry systems also restore degraded lands by making them productive through increasing N by biological nitrogen fixation (BNF), minimizing the emission of GHGs, and increasing carbon sequestration. BNF produces 300 kg N ha^−1^ year^−1^ and alley cropping produces 25 t ha^−1^ biomass that can be used to improve soil fertility and crop yields as biomass transfer (Kugedera, Mandumbu, and Nyamadzawo 2022). All these enhance agricultural resilience as farmers can grow a variety of crops in different areas (Afroz et al. 2025). Moreover, agroforestry systems enhance food systems as they improve the quality and quantity of products produced, such as vegetables from home gardens, sorghum, maize, and millets from open fields. Adoption of agroforestry was reported to increase yield in semi‐arid areas by approximately 50%–300% depending on farmer management. Sorghum and millets are common in dry lands and have been improved in their climate resilience with agroforestry to an extent where grain yield of sorghum was increased from 200 to 1100 kg ha^−1^ and even more in other countries like Kenya, India, and Uganda (Kimaru‐Muchai et al. 2021; Kugedera, Nyamadzawo, and Mandumbu 2022; Swadhi 2025). Agroforestry systems have the capacity to enhance agriculture for smallholder farmers in semi‐arid areas as shown in Table 1.

**TABLE 1:** Agroforestry systems that are compatible with smallholder farmers.

Agroforestry system	Effects	References
Biomass transfer	A cut and carry system that improve soil resilience, water retention, SOC, SOM, and crop growth. It has been reported to boost sorghum productivity in Kenya (190–4300 kg ha^−1^) and Zimbabwe (450–1146 kg ha^−1^) in sandy loam.	Kimaru‐Muchai et al. (2021) Kugedera, Nyamadzawo, Mandumbu, and Nyamangara (2022); Kugedera, Mandumbu, and Nyamadzawo (2022); Kugedera, Nyamadzawo, and Mandumbu (2022)
Alley cropping	Trees grown in rows and crops are also grown between tree rows where they are protected from high temperatures by shade. Foliage from trees act as mulch, decompose to release nutrients, increase infiltration of water, reduce emission of GHGs, increase soil resilience, crop growth, and yields.	Mantino et al. (2023)
Improved fallow	Improved fallow restores lost fertility, rehabilitates land, improves soil structure, resilience, and enhances crop yield by 45%–350% and food security by 20%–230% in semi‐arid areas.	Mafongoya et al. (2003) Munthali et al. (2014)

#### 3.2.4 Soil Water Conservation Strategies

Soil conservation improves soil resilience, minimizes nutrient loss, and enhances crop yields under the threat of climate change. These strategies include crop rotation, where rotating crops of different families and growth habits helps in minimizing soil erosion, recycling nutrients, and promoting sustainable crop productivity (Afroza et al. 2025). Enhanced water infiltration and retention are facilitated by soil conservation. Conservation of soil has the potential of reducing leaching by 45% and improving nutrient retention and moisture availability (Chitara et al. 2024). In addition, soil conservation strategies reduce surface runoff and soil erosion that may be caused by heavy rainfall and can also minimize chances of waterlogging and soil loss. An increase in water retention enhances crops' ability to withstand harsh conditions such as dry spells and increases crop resilience to drought (Raheem et al. 2025). Conservation agriculture mitigates climate change by lowering the use of synthetic fertilizers, increasing carbon storage, reducing emission of greenhouse gases, and enhancing resilience to climate change. Practices such as retaining crop residues increase soil moisture conservation, which helps decomposition and nutrient cycling that allow crops to survive nutrient stress and improve productivity. Water conservation techniques such as drip irrigation have the capacity to save water costs, allow farmers to grow a variety of crops throughout the season, and improve crop productivity, leading to food security for smallholder farmers (Ali et al. 2025). The systems enhance WUE that allows crops to survive and produce higher yields using little water (Arlanova et al. 2025). The conservation of soil and water is a key strategy in mitigating extreme temperatures and nutrient loss as this reduces leaching by almost 60%, increases soil moisture conservation by 23%–191%, and improves crop yield by more than 100%, especially for smallholder farmers in semi‐arid areas. Drip irrigation, for example, allows farmers to grow crops all year‐round, reducing the effects of erratic rainfall that contribute to food insecurity and low profitability.

Mulching is another water and soil conservation technique that reduces washing away of top soil, increases infiltration rates, and recharge soil moisture in the plant rooting zone so that crops survive and grow well during dry spell periods (Hakeem et al. 2025; Kayusi et al. 2025). Besides improving soil physiochemical properties, mulching reduces emissions of GHGs, increases soil carbon storage and crop yields (Ullah et al. 2022; Kayusi et al. 2025). Furthermore, mulching reduces evaporation of water directly from soil, improves soil health, and promotes quality crops. Mulching has been widely adopted and used by smallholder farmers in semi‐arid areas as a water conservation technology and reported to increase crop yields; for example, from 450 to 4200 kg ha^−1^ sorghum (Masaka et al. 2020), and 900 to 6500 kg ha^−1^ maize (Wu et al. 2025). A variety of crops can be grown under mulching including sorghum (Masaka et al. 2020), maize (Wu et al. 2025), soyabean and quinoa (Soomro et al. 2025; Wahidurromdloni et al. 2025).

Other water conservation techniques include tied ridges, tied contour, Zai pits, planting basins, and half‐moon. The techniques improve water retention in the soil and increase crop productivity, especially in low rainfall areas such as Chivi, Buhera, Marange, Mwenezi, and Matabeleland provinces (Mandumbu et al. 2021; Chiturike et al. 2024). Furthermore, they can be integrated with other options such as animal manure, agroforestry, compost, and mineral fertilizer to improve food systems in arid and semi‐arid areas (Kebenei et al. 2023; Debebe et al. 2025; Kubiku et al. 2025). Integrating tied contour with leucaena biomass transfer increased sorghum grain yield from 580 to 1146 kg ha^−1^ in Zimbabwe (Kugedera, Mandumbu, and Nyamadzawo 2022), and Zai pits integrated with *Tithonia diversifolia* biomass transfer increased sorghum grain yield from 190 to 430 Mg ha^−1^ in Kenya (Kimaru‐Muchai et al. 2021). Effects of these techniques are described in Table 2.

**TABLE 2:** Water conservation techniques and their effects on agricultural resilience and food systems.

Water conservation technique	Effects on agricultural resilience and food systems	References
Mulching	Provide surface cover that reduce soil erosion, surface runoff, and damage to soil structure. This increase soil resilience, infiltration of water, nutrient cycling, and boost crop growth. Mulching can increase crop yields by 100% or more. Minimize emission of GHGs thus lowering chances of climate change.	Masaka et al. (2020) Kayusi et al. (2025)
Half‐moon	Harvest rainwater, recharge soil moisture, allow crops to grow up to maturity with limited crisis of water stress. Increase crop yields and food systems in Burkina Faso and India.	Zougmoré et al. (2003) Kumar et al. (2024)
Zai pits	These are pits dug and crops are grown inside. They can be combined with organic or mineral fertilizer to increase soil resilience, health, crop growth, and yields. There are high chances of minimizing effects of climate change with the use of Zai pits and increase crop yield by 50%–350% in countries such as Kenya.	Kimaru‐Muchai et al. (2021) Kebenei et al. (2023)
Tied contour	These are mini‐dams that capture surface runoff, and rainwater along the field edge and hold it for long period so that it recharges soil moisture during dry spell. The technique increased grain yield of maize and sorghum in semi‐arid areas of Zimbabwe by 80%–500%.	Kubiku et al. (2022) Chiturike et al. (2024)
Infiltration pits	The pits are dug along the standard contour and they capture rainwater. Some farmers can construct them inside their fields to increase water availability during dry spell periods. Infiltration pits increased grain yield of sorghum and pearl millet in Zimbabwe and Tanzania by more than 1000 kg ha^−1^ and highest yield of 5675 kg ha^−1^ can be obtained from 250 kg ha^−1^.	Chilagane et al. (2020) Kugedera et al. (2023)

## 4 Conclusion

Low rainfall and soil infertility are the major constraints in dry regions and are mainly due to the changing climate. Changes in climate minimize chances of better food systems and resilience in agriculture. To mitigate effects of climate change, farmers can adopt practices such as biochar, mulching, water management, soil conservation, agroforestry, and drought‐tolerant crops to boost food systems and resilience in agriculture. Biochar, agroforestry, and mulching reduce emission of GHGs that cause climate change, increase conservation of water and soil, as well as improving crop productivity. The use of tied contour was seen as the major contributor in increasing soil moisture for sorghum as well as maize in developing Zimbabwe. Organic soil conditioner biochar has the capacity of increasing soil fertility, reducing leaching, and enhancing soil moisture that facilitates an increase in maize yield by 100%–300% in semi‐arid areas. Farmers in semi‐arid areas are recommended to adopt biochar as a soil conditioner, tied contour as an RWH technique, and sorghum/millets. RWH techniques have the potential of lengthening the cropping season and maximizing crop yields. So, their combination with organic amendments may help smallholder farmers to maximize yields and profits. Governments are also recommended to draft climate‐smart agriculture policies that support adoption and implementation of CSA practices to improve food systems in smallholder farming environments.

## Funding

The authors have nothing to report.

## Disclosure

AI declaration: The authors did not use any AI software.

## Conflicts of Interest

The authors declare no conflicts of interest.

## Data Availability

The authors have nothing to report.
